# The challenge of identifying species-rich taxa: Online multi-access key to Bornean *Cyrtandra* (Gesneriaceae)

**DOI:** 10.3897/BDJ.13.e143735

**Published:** 2025-03-10

**Authors:** Henna Tyyskä, Hannah Atkins

**Affiliations:** 1 Royal Botanic Garden Edinburgh, Edinburgh, United Kingdom Royal Botanic Garden Edinburgh Edinburgh United Kingdom; 2 Centro de Investigación Ecológica y Aplicaciones Forestales (CREAF), Bellaterra, Spain Centro de Investigación Ecológica y Aplicaciones Forestales (CREAF) Bellaterra Spain

**Keywords:** *Cyrtandra*, Gesneriaceae, multi-access taxonomic key, Borneo

## Abstract

Conservation and research of highly diverse plant taxa can be a considerable challenge due to unmanageable numbers of species with potentially complex relationships often resulting in difficulties in species identification. *Cyrtandra*, the largest genus of the family Gesneriaceae, exemplifies these challenges. The lack of identification resources for the ca. 170 species of Bornean *Cyrtandra* has left many specimens unidentified, slowing down the research efforts in the area. This project addresses this by constructing the first taxonomic key to all Bornean *Cyrtandra* and by describing the workflow of creating identification resources for highly diverse taxa, using the online biodiversity data management platform Xper3 (https://app.xper3.fr/). The key is now published and freely accessible online. Online multi-access taxonomic keys provide a promising tool for biodiversity research by combining an accessible user-friendly platform with dynamic tools for taxonomic research, making them particularly well suited for tackling highly diverse taxonomic groups.

## Introduction

Conservation of biological diversity remains one of the pressing challenges of the 21^st^ century. Borneo is the largest island in the Sundaland biodiversity hotspot (Myers et al. 2000), an area recognised as being a conservation priority because of the combination of high levels of endemism and also rapid habitat loss (Verma et al. 2020). Conservation actions require a good understanding of the species that are present to be effective. Large genera contribute significantly to this diversity, but are often poorly studied because their large size makes them challenging to tackle (Nic Lughadha et al. 2019, Atkins et al. 2021, Moonlight et al. 2024). A lack of taxonomic research and identification tools mean that many collections remain unnamed in herbaria around the world for many years, particularly in large groups (Bebber et al. 2010).

*Cyrtandra* is the largest genus in the family Gesneriaceae (Atkins et al. 2013) and represented in Borneo by ca. 170 species (Atkins et al. 2024). There is no recent revision of the genus on the island and information about the species is scattered in the literature. There is also no key to the species and very few illustrations, making it very challenging to accurately identify specimens to species-level. This project aims to address this by producing the first, illustrated, multi-access key to the 169 species of *Cyrtandra* in Borneo.

## Methods

### Taxonomy and terminology

All 169 currently recognised *Cyrtandra* species found in Borneo were included in this key (Atkins et al. 2024). The two subspecies and six varieties are currently not included, but users are provided with further resources for species with intraspecific classifications on the relevant species pages. We followed the taxonomic concepts and character terminology of Bornean *Cyrtandra* by O.M. Hilliard and B.L. Burtt who, together, described 116 out of the 169 recognised *Cyrtandra* species (Burtt 1970, Burtt 1978, Burtt 1982, Burtt 1990a, Burtt 1990b, Burtt 1993, Burtt 1997, Burtt 1999, Hilliard and Burtt 2002, Hilliard et al. 2003, Hilliard and Burtt 2004, Burtt 2005, Hilliard 2005, Hilliard and Burtt 2005a, Hilliard and Burtt 2005b, Hilliard and Burtt 2006a, Hilliard and Burtt 2006b) and several in regional works on Brunei (Burtt 1997) and Mt Kinabalu (Beaman et al. 2001).

**Figure 1.**
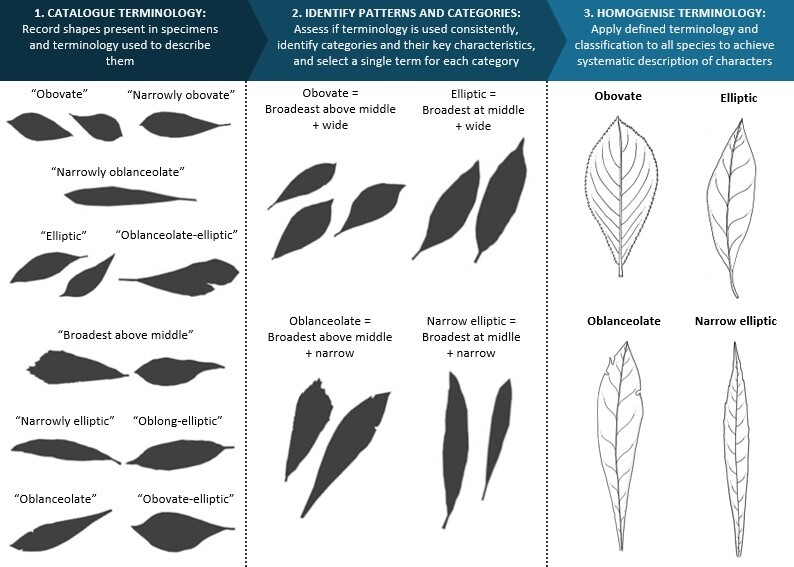
Workflow for homogenising botanical terminology across various source materials.

Botanical terminology can be subjective and used differently by different authors and within different taxonomic groups. This can lead to inconsistent descriptions of characters and a failure to correctly identify character states. To ensure consistent use of botanical terminology in this key, the species descriptions of Hilliard and Burtt were carefully studied to identify terminology used and morphological features associated with each term (Fig. 1). This terminology was then applied to species described by other authors to obtain consistent descriptions across species.

### Data

A character matrix was constructed to form the backbone of the key using all available data. The data sources used in this process were the original species descriptions, revised species descriptions by Hilliard and Burtt (Burtt 1970, Burtt 1978, Burtt 1982, Burtt 1990a, Burtt 1990b, Burtt 1993, Burtt 1997, Burtt 1999, Hilliard et al. 2003, Hilliard and Burtt 2004, Burtt 2005, Hilliard 2005, Hilliard and Burtt 2005a, Hilliard and Burtt 2005b, Hilliard and Burtt 2006a, Hilliard and Burtt 2006b), Hilliard’s unpublished illustrations of floral morphology of each *Cyrtandra* species, type specimens and further specimens identified by Hilliard and Burtt at the Royal Botanic Garden Edinburgh. Herbarium specimens identified by other authors were not included to stay consistent with the species concepts of Hilliard and Burtt.

### Characters and character states

The final set of characters used in the key was selected, based on taxonomic significance, discriminatory power and user-friendliness. Characters that were taxonomically insignificant (consistent across all species), highly subjective to describe, difficult to accurately assess or had limited data availability were eliminated from the dataset. The final dataset includes both geographic and morphological characters (Table 1). Most morphological characters can be easily observed on both living and herbarium materials, with a few characters of floral morphology requiring a 10x hand lens, microscope or a dissection kit to assess. Each species was scored for all characters for which the data were available. Some characters, such as the leaf shape, show considerable intraspecific variation and these characters were scored for all applicable character states to reflect the morphological diversity.

The character states within each character were carefully assessed and defined to clearly communicate the variation present. This included the definition of boundaries between character states, addition of character states to reflect morphological variation and reduction of complicated character states into more clearcut, broad categories. For example, the botanical terminology used to describe the shape of the bracts is highly inconsistent within the literature and the shapes do not form clear natural categories. Consequently, the relative length and width of the bracts (length to width ratio) was used as a proxy for bract shape to create consistent and objective categorisation.

While the absolute measurements of various organs can be a useful and objective character to record, it is difficult to estimate the amount of intraspecific variation present in a poorly-known species. Measurements presented in protologues and currently known herbarium specimens offer a good indication of the general size of plant organs, but do not necessarily capture the degree of variation present in natural populations. Vegetative characters appear particularly variable in certain *Cyrtandra* species. To take this into account, measurements were used in a very conservative manner as broad categories with the aim of filtering out the species with unusually large or small organs.

### Xper3 key construction

The final data matrix of the species scored for their character states was exported into the Xper3 platform (Vignes-Lebbe et al. 2016). The descriptive model was finished by adding detailed descriptions and illustrations for each character and character state to aid with identification. Characters were then manually assigned a weight between 1 to 5 to reflect their taxonomic significance, which prompts the programme to present these characters at the top of the key. Using the descriptive model and species data, Xper3 will automatically create a multi-access identification key.

### Illustrations

Illustrations were provided as a visual aid for accurately interpreting characters and character states, as well as to support correct identification of species. Original illustrations were used where available; however, most Bornean *Cyrtandra* species were not published with illustrations. The remaining illustrations consist of photographs sourced from Royal Botanic Garden Edinburgh (RBGE) archives, photographs provided by *Cyrtandra* experts and collaborators, photographs of *Cyrtandra* growing in RBGE living collections and herbarium specimens, Hilliard’s unpublished illustrations of floral morphology (adapted and rescaled) and original illustrations, based on living materials, herbarium specimens and photographs. Having expert-verified illustrations for each species is crucial for confirming the identification. In groups where there are potentially undescribed species, the extra step of confirming the determination is essential.

### Testing

The key was tested by the following user groups: the key authors, *Cyrtandra* experts and inexperienced users without expertise in *Cyrtandra*. The testing was conducted by participants using the key on a set of photographs taken in the field and of herbarium specimens. The participants recorded their identification results, as well as feedback on the usefulness and user experience on various aspects of the key. Based on this feedback, some of the character descriptions were updated and some ineffectual characters were removed to promote more accurate identification process.

## Results

The finished key is available online at https://cyrtandra-borneo.identificationkey.org/. A user manual is provided in the supplementary materials of this publication (Suppl. material 1).

## Discussion

### Benefits of multi-access identification tools

There is a need for user-friendly taxonomic resources to facilitate biodiversity research within various scientific disciplines (Gotelli 2004). Online multi-access keys can answer this need by providing globally available open-access identification materials with features that make them particularly well-suited for non-specialist users. Online platforms allow easy incorporation of hyperlinks and multimedia, which can enrich the descriptions provided and facilitate knowledge sharing. Additional materials, such as detailed descriptions of specialist terminology used, are particularly important for the creation of user-friendly taxonomic resources and can be easily included in online materials.

Multi-access keys have several benefits over traditional dichotomous keys. Firstly, multi-access keys allow users to start the identification process at their character of choice, rather than following a set list of questions in a linear fashion. For example, characteristics of reproductive organs (flowers and fruits) usually bear high diagnostic significance in plants and, consequently, they are widely used in taxonomic keys. However, due to the seasonal and ephemeral nature of these organs, they can often be missing from specimens. Multi-access keys allow users to avoid getting stuck with questions they cannot answer due to the absence of specific characters in their data by allowing them to choose which questions to answer. This can also increase the accuracy of the identification by allowing preferential use of characters which the user can identify with high confidence and which can be particularly beneficial for non-specialist users.

Based on the questions the users choose to answer, this process may not result in species-level identification. However, in the Xper3-platform, it is possible to constantly see the names and characteristics of the remaining species while using the key, which allows the user to easily manually assess the remaining species. In our key, this includes additional information such as illustrations and hyperlinks to the type specimens that are available online, which can support the identification process beyond the scored data.

The non-linear process also allows users to select multiple character states at any point. While this increases the number of questions required for identification, the lack of mutually exclusive selection facilitates the incorporation of highly variable characters, as taxonomic units do not need to fit to a single character state. In *Cyrtandra*, for example, leaf shape can either be relatively constant within species or show a high degree of intraspecific variability. By scoring a species’ leaf shape for all matching shapes, the diversity can be represented without having to create a separate category for each unique combination of shapes. Thus, multi-access keys offer an alternative to traditional dichotomous keys and may be particularly well suited as a tool for studying diverse taxonomic groups and for creating accessible user-friendly resources.

### Taxonomic keys as tools for studying plant diversity

Highly diverse taxonomic groups such as large plant genera can present considerable challenges to taxonomists because of the unmanageable numbers and potential for complex relationships between species due to rapid diversification and they have often been neglected in the past for this reason (Frodin 2004, Moonlight et al. 2018, Atkins et al. 2021). It is anticipated, however, that new methods and data availability combined with global collaborations may make it more feasible to tackle these large groups (Moonlight et al. 2024).

The process of constructing taxonomic keys traditionally heavily relies on having a well-resolved taxonomy of the target group. This may not be realistic for many highly diverse taxa due to the limits of our current knowledge, which may create the question of the usefulness of identification resources in such groups. Here, we argue that an identification key in the absence of a well-resolved taxonomy can be a stepping stone in taxonomic research, as it allows easy comparison between species and faster identification of specimens, in the context of our current taxonomic understanding of them. By systematically checking all species names in a group, a key can also aid in recognising undescribed species and provide a foundation for their formal description. However, we emphasise that, without a well-resolved taxonomy, the purpose of an identification key needs to be redefined and the key construction must clearly identify the source information of its taxonomic backbone. In this key, for example, the taxonomy is based on the species concepts of Hilliard and Burtt.

When used conservatively, a multi-access taxonomic key can be an excellent aid in research. The creation of such a key itself collates a dataset of taxonomically informative characters. In the Xper3 platform, the authors of a key can access other functions, such as comparing different pairs or groups of species using these data. The key can be used to rapidly narrow down species based on characters of interest, without necessarily attempting to identify specimens to species level. In a genus with more than 100 species, narrowing down to a manageable number will still significantly speed up the process of identification.

Finally, the Xper3 platform allows updating the dataset as new information accumulates, which is a useful feature in a group in which new discoveries are expected. The species delimitations or descriptions of our key may change in the future to reflect this progress in taxonomic research, making it a dynamic tool for both research and identification.

## Conclusion

As outlined in the Introduction, herbaria contain large numbers of unidentified specimens, particularly in species-rich taxa. Accurately naming these collections is a huge contribution to our understanding of the distribution and taxonomy of species in these groups. In these understudied groups where the taxonomy is not complete and there may still be significant numbers of undescribed species, these keys also have potential as a tool for highlighting specimens that cannot be identified which can be useful for taxonomic researchers in these groups.

## Supplementary Material

D2622DBE-84FF-57E9-B2F2-E6CD7743B58C 10.3897/BDJ.13.e143735.suppl1 Supplementary material 1 Cyrtandra of Borneo - User Manual Data type User manual (PDF) Brief description User guide for navigating the Xper3 interphase. File: oo_1247893.pdf https://binary.pensoft.net/file/1247893 Tyyskä & Atkins

## Figures and Tables

**Table 1.** List of characters used in the identification key.

**Character name**	**Character type**
Geographical region	Categorical
Elevation (m)	Numeric
Growth form	Categorical
Leaf arrangement	Categorical
Leaf length (mm)	Numeric
Leaf width (mm)	Numeric
Petiole length (mm)	Numeric
Leaf shape	Categorical
Leaf apex shape	Categorical
Leaf base shape	Categorical
Leaf attachment	Categorical
Leaf serration	Categorical
Leaf upper surface hairs	Categorical
Leaf upper surface: mamillate	Categorical
Leaf upper surface: vermiform sclereids	Categorical
Bract type	Categorical
Bract length (mm)	Numeric
Bract margins	Categorical
Bract hairs	Categorical
Bract dimensions	Numeric
Bract glands	Categorical
Pedicel length (mm)	Numeric
Calyx shape	Categorical
Calyx length (mm)	Numeric
Calyx hairs	Categorical
Corolla base colour	Categorical
Corolla length (mm)	Numeric
Corolla external hairs	Categorical
Corolla internal indumentum	Categorical
Stamen indumentum	Categorical
Disc type	Categorical
Ovary indumentum	Categorical
Coma	Categorical
Fruit length (mm)	Numeric
Fruit shape	Categorical
Fruit texture	Categorical
