# Supplementary material for: The challenge of identifying species-rich taxa: Online multi-access key to Bornean Cyrtandra (Gesneriaceae)
Source: Biodivers Data J. 2025 Mar 10;13:e143735. doi: 10.3897/BDJ.13.e143735 (PMC11915011; doi:10.3897/BDJ.13.e143735)
Supplement: Supplementary material 1 — Cyrtandra of Borneo - User Manual [file bdj-13-e143735-s001.pdf]

Cyrtandra of Borneo

Tyyskä, H. & Atkins, H.J.

Cyrtandra of Borneo is an online multi-access taxonomic key to species of *Cyrtandra* (Gesneriaceae) found on the island of Borneo. The key is hosted by the Xper3 platform (https://www.xper3.fr) and it is publicly available at https://cyrtandra-borneo.identificationkey.org/

User Guide:

A. Click characters in the *Descriptors*-tab to view the character states associated with each character.
B. You can also search for characters using the *search bar* at the bottom of the window.
C. Tick boxes to choose character states that match your specimen. You can select multiple character states if applicable. The number associated with each character state indicates the number of species left after the selection.
D. You can click *unselect* (or simply click the same character again) to undo your current selection.
E. Click *submit* to confirm your selection.
F. Submitting your selection will update the *Remaining taxa*-column. The column lists species based on how well their characters match your selection. You can click the species in this column at any time to learn more about them. The length of the green bars indicates the percentage of selected characters that match each species.
G. Click *History*-tab to see and modify character states you have selected.
H. Click these symbols to either delete or modify your past selections.
I. To start over and clear the entire selection, click the *double arrow symbol* in either *Descriptors*- or *History*-tab.

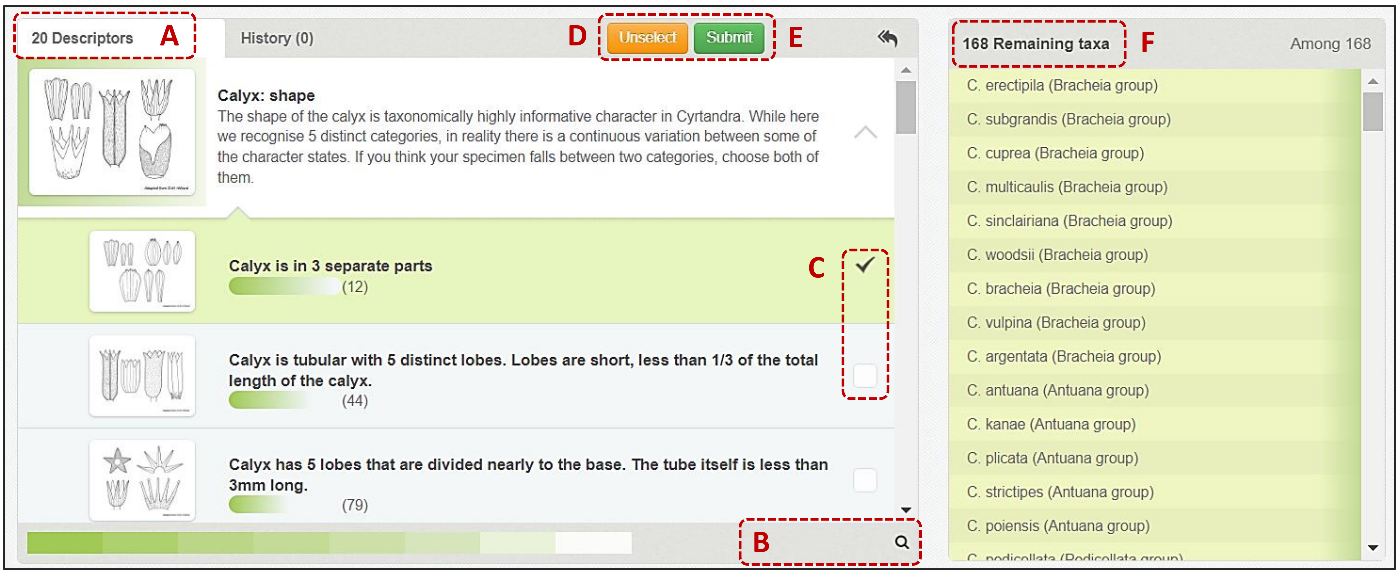

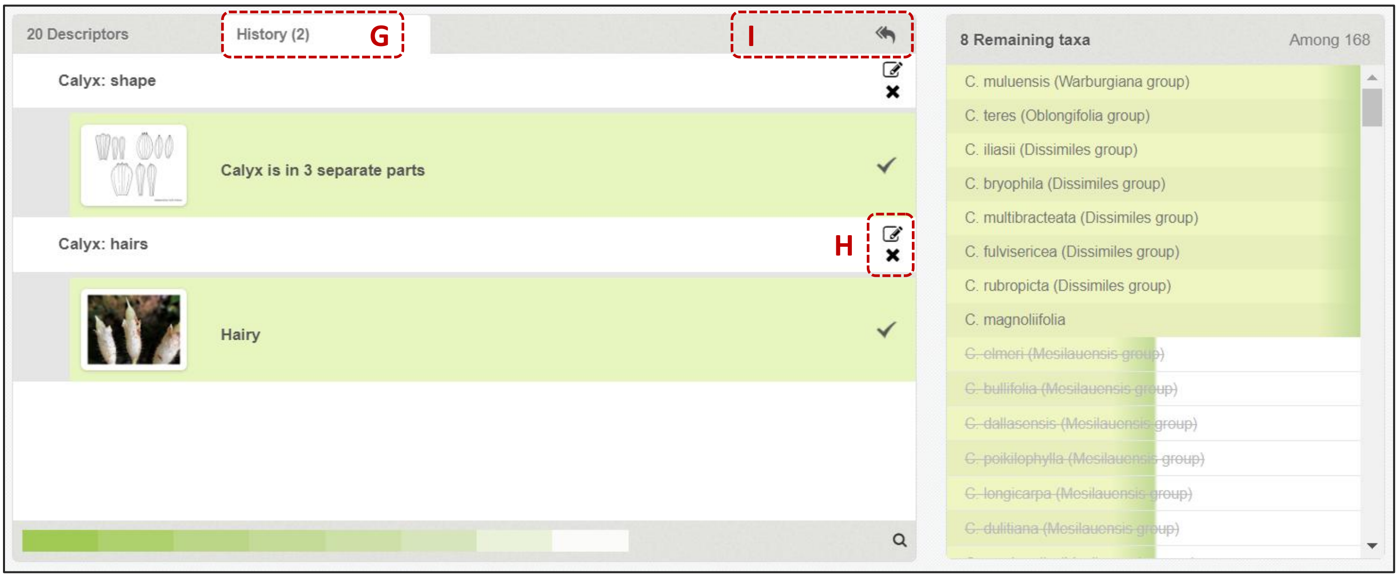

Additional Tips:

- ✓ If your specimen matched multiple character states or appears to be between character states, choose both to avoid ruling out species that could be a match.

- ✓ Some characters are more taxonomically informative than others and the key is designed to reflect this: these key characters appear at the top of the *Descriptors*-list.

- ✓ If your specimen has a rare character, it can often be identified faster. Some of the rare characters to look for include: Rosette leaf arrangement, mamillate leaf surface, vermiform sclereids at the leaf surface, the presence of a coma at the top of the ovary, and smooth fruit surface.

- ✓ When you click species in the *Remaining taxa*-column, in the notes section you will find **a reference to the protologue** of that species and **a link to the type specimen** (if available online).
